# Subliminal Face Emotion Processing: A Comparison of Fearful and Disgusted Faces

**DOI:** 10.3389/fpsyg.2017.01028

**Published:** 2017-06-21

**Authors:** Shah Khalid, Ulrich Ansorge

**Affiliations:** ^1^Institute of Cognitive Science, University of OsnabrückOsnabrück, Germany; ^2^Faculty of Psychology, University of ViennaVienna, Austria

**Keywords:** face emotion processing, priming, subcortical, disgust, fear

## Abstract

Prior research has provided evidence for (1) subcortical processing of subliminal facial expressions of emotion and (2) for the emotion-specificity of these processes. Here, we investigated if this is also true for the processing of the subliminal facial display of disgust. In Experiment 1, we used differently filtered masked prime faces portraying emotionally neutral or disgusted expressions presented prior to clearly visible target faces to test if the masked primes exerted an influence on target processing nonetheless. Whereas we found evidence for subliminal face congruence or priming effects, in particular, reverse priming by low spatial frequencies disgusted face primes, we did not find any support for a subcortical origin of the effect. In Experiment 2, we compared the influence of subliminal disgusted faces with that of subliminal fearful faces and demonstrated a behavioral performance difference between the two, pointing to an emotion-specific processing of the disgusted facial expressions. In both experiments, we also tested for the dependence of the subliminal emotional face processing on spatial attention – with mixed results, suggesting an attention-independence in Experiment 1 but not in Experiment 2 –, and we found perfect masking of the face primes – that is, proof of the subliminality of the prime faces. Based on our findings, we speculate that subliminal facial expressions of disgust could afford easy avoidance of these faces. This could be a unique effect of disgusted faces as compared to other emotional facial displays, at least under the conditions studied here.

## Introduction

From a phylogenetic perspective, the quick and effortless recognition of human emotional facial expressions provided an evolutionary benefit: A high ability to recognize fearful or disgusting faces, for example, would have increased human sensitivity to potentially harmful threats in the environment that, if avoided, would have increased inclusive fitness (Whalen, 1998).

In line with this reasoning, several studies provided evidence of even subliminal processing (i.e., processing of stimuli presented below the threshold of awareness) of human emotional expressions (Whalen et al., 1998; de Gelder et al., 1999; Morris et al., 1999; Dimberg et al., 2000; Kiss and Eimer, 2008; Smith, 2012). For example, using electroencephalography (EEG), Smith (2012) studied the time-course of processing of fearful, disgusted, happy, and neutral facial expressions. The participants were asked to categorize the masked faces for their expressions. The author found differences in activity patterns in the frontal and occipito-temporal brain regions, where visual backward masking by a stimulus following a face displaying an emotion prevented the face’s visibility. What is particularly noteworthy about Smith’s study is that, although two types of masked faces contained negative expressions, there were some processing differences between these faces: The masked fearful faces produced greater activation in the frontal region when compared to the masked disgusted faces. Such EEG differences are impressive as they indicate that, even if not registered consciously, two facial expressions of different emotions but of the same negative affective valence recruit different brain areas, ruling out that negative (vs. positive) valence of the affect accounted for the EEG differences and supporting instead an interpretation in terms of emotion-specific processing (see also, e.g., Willenbockel et al., 2012).

In the current study, we picked up on this emotion-specificity to look closer into the processing of disgustedly looking faces (Experiments 1 and 2) and to compare their processing to that of fearful faces (Experiment 2). Based on the known differential sensitivity of human subcortical and cortical visual processing pathways to high-spatial frequency (HSF) content, in Experiment 1, we tested if subliminally presented disgusted faces could probably be processed by the subcortical route, as suggested by more or less related prior research showing evidence of subcortical processing of emotional facial expressions (Pegna et al., 2005; Leh et al., 2006; Willenbockel et al., 2012; for a more general argument, see also LeDoux, 1996, 1998). To that end, we conducted a masked priming experiment, with masked emotional faces as primes and visible faces as targets (Morris et al., 1999; Finkbeiner and Palermo, 2009). In each trial of the experiment, participants judged if the clearly visible target face that they saw was a neutral or a disgusted face. In addition, unknown to the participants, a masked face of the same or of a different emotional expression was presented before each target face. In congruent conditions, displayed emotions of prime and target were the same (e.g., both were disgusted faces), while in incongruent conditions, they were different (e.g., a disgusted face prime preceded a neutral face target). Typically, when comparing between the two conditions, one can expect a priming or congruence effect, with better performance in congruent than incongruent conditions (cf. Neumann and Lozo, 2012). Furthermore, one can also expect to see little or no evidence for the ability to discriminate between different primes in the same participants that show the priming effect. In the current study, this latter prediction was tested in a separate prime visibility test at the end of each experiment.

Importantly, to test a potential subcortical origin of the expected priming effect, in Experiment 1, we used high-pass filtered (HSF) faces as primes for half of our participants and low-pass filtered (LSF) faces as primes for the other half, where LSF face primes would be processed by both, cortical and subcortical, processing routes, and where HSF face primes would only be processed by the cortical processing route (Khalid et al., 2013). We hypothesized that, if subcortical processing accounted for the expected priming effects, priming effects should be stronger or selectively present with unfiltered primes (i.e., having a full band of frequencies) and low-spatial frequency (LSF) primes (i.e., with LSF primes) but not with HSF primes (i.e., with HSF-pass filtered primes).

Experiment 2 tested if the priming effect of Experiment 1 was indeed due to emotion-specific processing. To that end, we included fearful and neutral faces in one block of trials and disgusted and neutral faces in the other block of trials, and we looked for differences between the priming effects of the different blocks. These were to be expected if the priming effects reflected emotion-specific processing (here: of disgust vs. fear). (Of course, in case that the priming effects of disgusted and fearful face primes were the same, they would not necessarily have been also the same in terms of the underlying brain processes. However, at least, if a difference in the priming effects was found, that would have supported their different origins.) In contrast to Experiment 1, only non-filtered face primes were used in Experiment 2, allowing us to investigate if the unexpected sign of the priming effect of the disgusted face primes (that we found in Experiment 1) owes to the fact that the face primes were filtered.

Finally, in both experiments, we also tested the influence of attention (here, of spatial cueing) on priming effects. To that end, face primes were presented slightly above and targets slightly below screen center, so that a visual cue temporally preceding the prime could direct the participants’ attention either toward the prime location or toward the target location (Finkbeiner and Palermo, 2009). In this way, we were able to investigate if the priming effect depended upon spatial attention. If the priming effect depended upon spatial attention, we expected to see stronger or selective priming effects in prime-cued conditions as compared to target-cued conditions, but in past research it was found that, under relatively similar conditions (though with a different task and different faces), face priming effects were independent of attention (i.e., of cue position), supporting the classical notion that at least some subliminal processes operate independently of attention (Posner and Snyder, 1975; Finkbeiner and Palermo, 2009).

## Experiment 1

In this experiment, we tested whether the emotional facial expression of disgust can be processed subliminally and whether such processing follows the subcortical route. Participants categorized visible target faces as either displaying a neutral or a disgusted facial expression. The target face was preceded by a masked prime face in a prime-target congruent (both faces displaying the same emotional expression) or incongruent sequence (both faces displaying different emotional expressions). The face primes were unfiltered, HSF (for half of the participants) or LSF (for the other half of the participants). The target faces were unfiltered. In the visual display, the primes were presented slightly above and the targets below screen center. In order to shift participants’ attention, a cue was presented before the prime, either at the prime’s location (in the prime-cued condition) or at the target’s location (in the target-cued condition; Finkbeiner and Palermo, 2009; Khalid et al., 2013, 2015).

If processing of the subliminal disgusted face’s occurs only along the subcortical route, we expected a priming effect for the LSF primes and the unfiltered primes because this route has been shown to be sensitive to the LSF content of visual signals in general (Schiller et al., 1979) and to emotional expressions in particular (Vuilleumier et al., 2003). The HSF primes would then simply fail to produce a priming effect because of their lack of influence on the subcortical route (cf. Khalid et al., 2015, 2013). However, if subliminal face processing reflects processing along the primary retino-geniculate projection, no such dependence of the priming effect on LSF content should be observed and the priming effect should be found with HSF primes, too, because the retino-geniculate projection is sensitive to this type of visual input.

### Method

#### Participants

Forty students (27 female) with a mean age of 23.1 years participated. Measurement by the German version of the Questionnaire for the Assessment of Disgust Proneness (QADP; Schienle et al., 2002) showed that all participants had normal sensitivity (*M* = 2.0, *SD* = 0.4) level. Half of the participants were tested in the HSF group and the other half in LSF group. All participants in the current study had normal or corrected to normal vision, gave their informed consent, were treated in accordance with APA standards and the rules of the declaration of Helsinki, and received course credit in exchange for their participation. For the current study, we obtained ethical approval from the ethics committee of the University of Osnabrueck, Germany, where the study was conducted.

Using G^∗^Power (Faul et al., 2007), the a-priori power analysis for the main effect of congruence, from the effect size *f* = 0.61 achieved in an earlier study of Khalid et al. (2013), showed that a required sample size of *n* = 32 would have been needed to obtain statistical power (1 – β) at the 0.95 level.

#### Apparatus

Visual stimuli were presented on a 17-inch, color flat screen display, with a refresh rate of 59.1 Hz, steered by an NVIDIA GeForce GT 220 (with 1024 MB) graphics adapter. Accurate timing of the display was verified by repeatedly presenting the stimuli for their intended duration one by one and recording their duration through a cathode ray oscilloscope. We found that in all cases the precision of presentation duration was better than 1 ms.

Participants sat at a distance of 57 cm from the screen in a quiet, dimly lit room, with their head resting in a chin rest to ensure a constant viewing distance and a straight-ahead gaze direction. Reaction times (RTs) were registered via a standard serial computer keyboard, placed directly in front of the participants. Target responses were given through the keys ‘C’ and ‘M’ (covered and labeled as ‘left’ and ‘right,’ respectively). At the beginning, participants placed their left and right index fingers on the appropriate keys. After reading the instructions, the participants pressed the spacebar with one of their thumbs to start the experiment. Pressing the space bar was required prior to each trial, so that the participants could also take breaks at their convenience by simply not pressing the bar.

#### Stimuli

We used the forward mask and cues of previous studies (Finkbeiner and Palermo, 2009; Khalid et al., 2013, 2015). The forward mask was a checkerboard pattern. The backward mask was a scrambled composite of all face images used. The face targets and primes wore neutral or disgusted emotional expressions. All face images in the current study were originally taken from the Karolinska Directed Emotional Faces (KDEF) database (Lundqvist et al., 1998). All of the face stimuli were cropped in an oval layer so that only the face features were presented. Each image subtended a visual angle of 3.0° vertically and 2.5° horizontally. All face stimuli, here as well as in the following experiment, were equated for luminance and contrast (mean root mean square contrast = 8.39, *SD* = 0.05), as well as spectral power (mean amplitude = 6.63, *SD* = 0.48).

**FIGURE 1:**
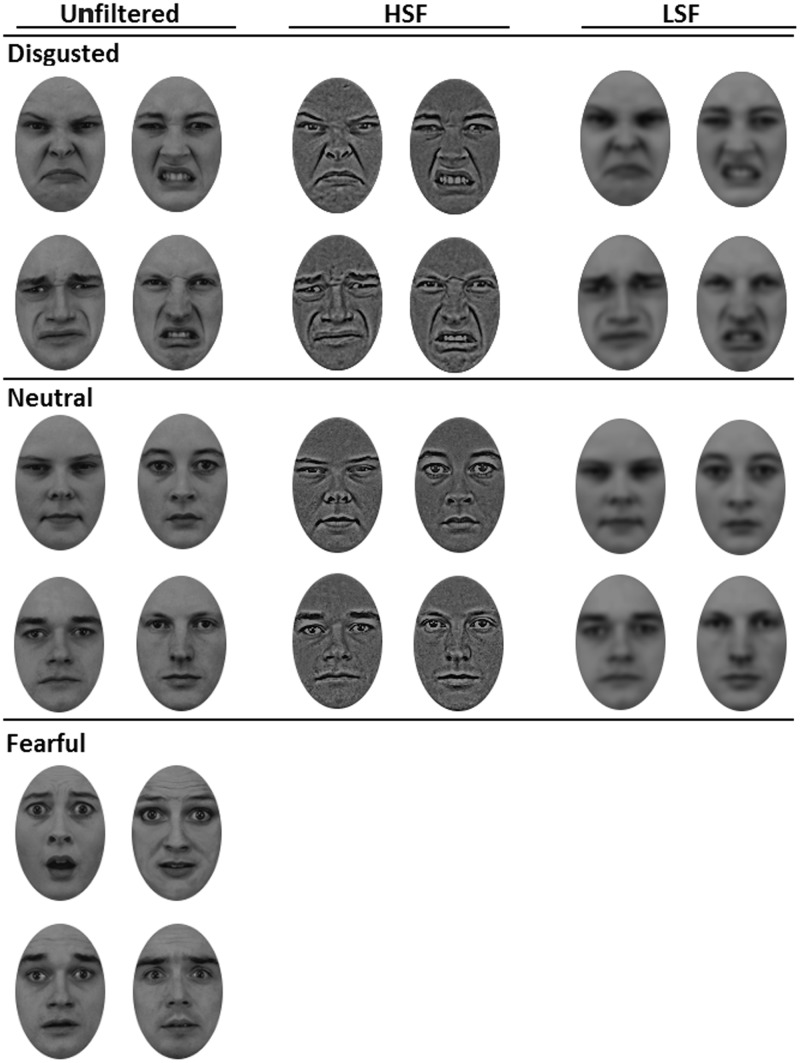
Set of female and male face targets and primes used in the current study. From left to right: unfiltered face primes and targets used in Experiments 1 and 2, high-pass filtered (HSF) face primes used in Experiment 1 (Neutral vs. Disgusted) and low-pass filtered (LSF) face primes also used in Experiment 1 (Neutral vs. Disgusted). All faces were equated for luminance and contrast (mean root mean square contrast = 8.39, *SD* = 0.05), as well as spectral power (mean amplitude = 6.63, *SD* = 0.48).

The face targets and primes were selected on the basis of participants’ performance in a pilot experiment. In the pilot experiment, six male and six female face images in each fearful and disgusted category were presented to 26 participants (18 female, mean age = 21.8 years) using the same procedure as for the current study, except that the masks, primes, and cues were replaced with blank screens, thus, showing only the clearly visible face targets. Participants were asked to categorize the face targets as disgusted or being of a different emotion. On the basis of the participants’ performance (RTs and error rates [ERs]), four disgusted and four neutral faces, two male and two female individuals (mean RTs = 604 ms, mean ERs = 11.8%), were selected as primes and targets for the current study (see **Figure 1** for the faces). Targets and primes were similar, except that half of the primes were either HSF or LSF as shown in **Figure 1**.

#### Procedure

**FIGURE 2:**
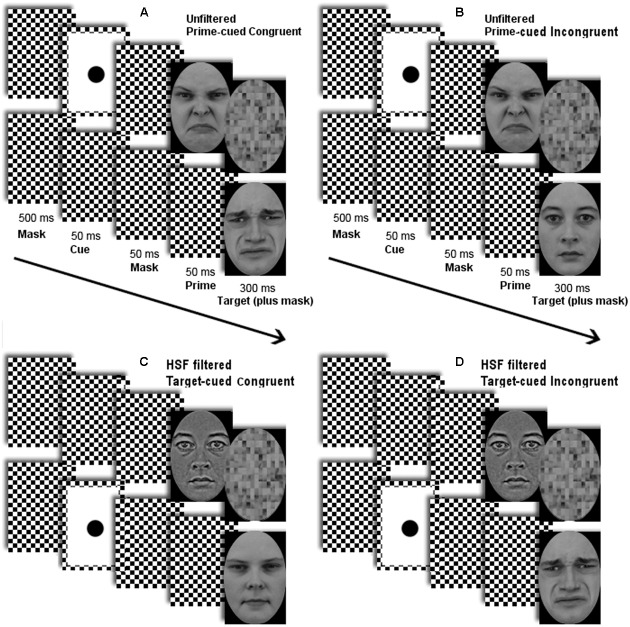
Depicted are sequences of stimuli with **(A)** an unfiltered face prime in a prime-cued disgusted-target congruent trial, **(B)** an unfiltered face prime in a prime-cued neutral-target incongruent trial, **(C)** a high-spatial frequency (HSF) filtered face prime in a target-cued neutral-target congruent trial, and **(D)** an HSF filtered face prime in a target-cued disgusted-target incongruent trial of Experiment 1. (The same conditions were used in Experiment 2, but with fearful primes and targets in half of the trials.) Arrows depict the flow of time.

See **Figure 2** for examples of sequences of events in a trial. Stimuli were presented on a black screen (luminance < 0.1 cd/m^2^). In each trial, two streams of stimuli were presented, one stream directly above the screen center, the other directly below it, with the target always at the lower location and the prime always at the upper location (Finkbeiner and Palermo, 2009; Khalid et al., 2013, 2015).

Each trial began with a checkerboard mask for 500 ms. Next, a non-predictive cue was shown for 50 ms at either prime or target location, together with the checkerboard mask at the non-cued location. The cue was used to capture attention toward the prime (in the prime-cued condition) or toward the target (in the target-cued condition). After this, the checkerboard mask was shown for 50 ms, again at both locations, followed by the prime face at the upper location together with the checkerboard mask at the target’s location for 50 ms.

All face stimuli were used both as primes and targets, but half of the primes were LSF for one group and HSF for another group of participants. To avoid repetition priming, in each trial, the same face was never shown as both prime and target (Forster and Davis, 1984; Norris and Kinoshita, 2008). In the final frames of a trial, the target face was presented at the lower location for 300 ms together with a scrambled-face backward mask at the upper (prime) location.

Across trials, emotional expressions of prime and target varied orthogonally to create congruent and incongruent conditions. In the congruent condition, the prime face’s emotional expression was the same as that of the target face: A disgusted face prime was presented prior to a disgusted face target, or a neutral face prime was presented prior to a neutral face target. In the incongruent condition, the prime face’s emotional expression was different from that of the target face: A disgusted face prime was presented prior to a neutral face target, or a neutral face prime was shown prior to a disgusted face target.

In the target-discrimination task, participants had to discriminate the emotional expression of the target face as either neutral or disgusted by pressing one of the assigned buttons (counter balanced across participants) as quickly and as accurately as possible. Slow responses (RTs > 850 ms) and errors were discouraged by slightly delaying feedback displays presented for 750 ms, reading ‘respond faster!’ and ‘wrong key!,’ respectively. The target-discrimination task consisted of two within-participant blocks, one with unfiltered primes and the other with filtered primes, the order of which was counter balanced across participants. Each block consisted of 32 repetitions of each combination of cueing (prime-cued, target-cued), target type (disgusted, neutral), and prime-target congruence level (congruent, incongruent), for a total of 256 trials, thus, a total of 512 trials for the target-discrimination task. In the filtered primes block, we also included 10% filler trials (not included in the analysis) in which the HSF and the LSF primes were presented as targets. These filler trials were randomly intermixed with the unfiltered face target trials to ensure that participants did not search only for the unfiltered face targets but also for the HSF and LSF content (cf. Ansorge et al., 2010a; Khalid et al., 2013). An additional 32 training trials were included at the start of each block.

At the end of the experiment, participants performed a block of prime-discrimination trials, in which they had to categorize the masked primes. For this prime-discrimination task, participants first categorized the visible target (just as they had in the target-discrimination task), and then they categorized the masked prime. The same emotion-to-response-button mapping was used for the prime faces as for the target faces (cf. Finkbeiner and Palermo, 2009; Khalid et al., 2013, 2015). The prime-discrimination task also consisted of two blocks, one with unfiltered primes and one with filtered primes, with 128 trials per each block, thus, a total of 256 trials. Analogously to the target-discrimination block, the filtered primes block additionally contained 10% trials with the filtered primes as targets (excluded from analysis).

Within each block, the different conditions were presented in a pseudo-random sequence, with the two constraints that no particular face target was repeated in immediately succeeding trials and that no more than four trials in a row required the same target response. The experiment started with the target-discrimination block, followed by the masked prime-discrimination block.

At the very end of the experiment, we included one further block, with unmasked face primes of 50 ms duration at their upper position only. This final block was included to verify that the face prime alone contained sufficient information to allow successful emotional expression discrimination, at least under supraliminal (unmasked) conditions. In this final block, all of the masks (checkerboards and composites of scrambled faces) and the targets were left out (i.e., all these stimuli were replaced by blank screens), and the participants were asked to discriminate between the emotional expressions of the unmasked prime faces. This block consisted of a total of 80 trials, 40 trials with unfiltered primes and further 40 trials with filtered primes.

The experiment was run in a single session, with eight short breaks, one in the middle and one at the end of each of the four within-participant target-discrimination and prime-discrimination blocks. The whole experiment took approximately 40 min.

Besides the major questions of Experiment 1, we also wanted to test the temporal development of the priming effect. This was done by sorting RTs from fast to slow responses and dividing the ordered distribution into three roughly equally sized bins per each condition of interest. Due to this binning procedure, we were able to look at the differences between congruent and incongruent conditions as a function of the RT bin. For example, priming effects can sometimes be short-lived and are only evident among the fastest responses, so that with a more conservative response criterion, if more time passes since the prime has been presented, less priming effects can be observed (Kinoshita and Hunt, 2008; Ansorge et al., 2010b). However, one should note that this binning procedure has two potential drawbacks. Firstly, although according to Ratcliff (1979), 10 trials are sufficient per each mean in vincentizing, in the current study, this procedure indeed left only a small number of trials per bin and condition (i.e., 10.67). Secondly, binning did not allow for perfect counterbalancing with the other experimental factors because of the fractional number of trials per bin and because of not replacing outliers and errors. (The latter, however, is often the case in vincentizing.) However, slightly different numbers of trials for the different levels of the same independent variables are standard in psychological research because different error rates for different levels of independent variables typically jeopardize equal numbers of trials per each level of each variable anyway. Also, similar approaches – inclusion of an additional variable “bins” after an initial analysis – have been taken in other research areas with the aim of scrutinizing the conclusions of an initial analysis once more. For instance, with the help of a median split of the RTs, McDonald et al. (2013) showed, contrary to initial conclusions of Hickey et al. (2006), that interference effects of irrelevant distractors were restricted to the slowest responses. As an aside, we noted that McDonald et al. also used a larger sample size than Hickey et al. to overcome power issues of the former study. This, however, was not recommended in the case of the present study as our analysis indicated sufficient power.

### Results

#### Target-Discrimination Task

**FIGURE 3:**
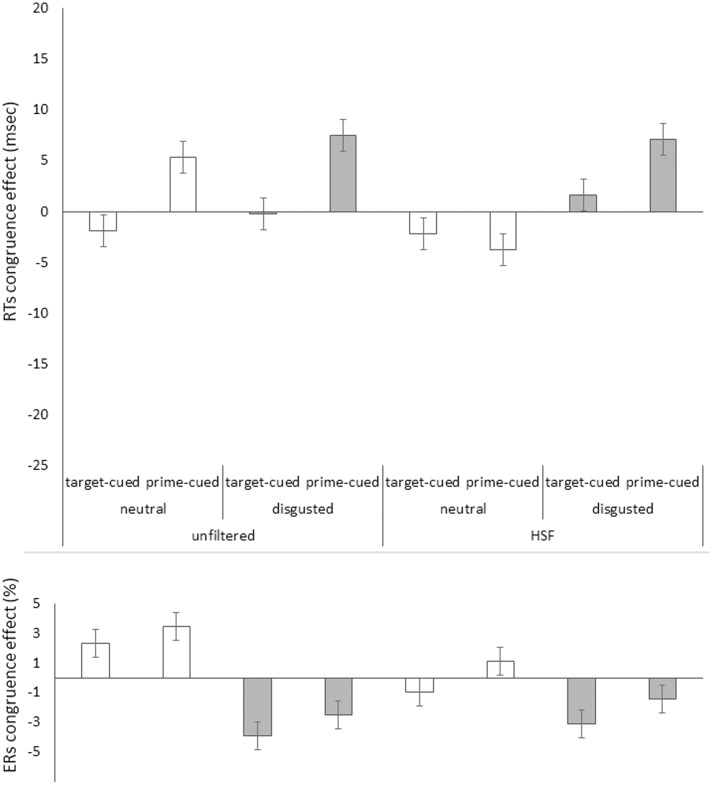
Priming effects (incongruent RTs minus congruent RTs) in mean correct Reaction Times (RTs) in milliseconds **(Upper)** and in error rates (ERs) in percent **(Lower)** on the *y* axis plotted as a function of prime face filtering (unfiltered vs. HSF filtered primes), target face valence (neutral [white bars] vs. disgusted [gray bars]), and cue type (target-cued vs. prime-cued) in Experiment 1’s HSF block on the *x* axis. Jointly, the congruence effects in RTs and ERs show positive priming effects in the neutral faces’ prime-cued condition, and reversed priming effects in the disgusted faces’ target-cued condition. All other conditions apparently show no effects or evidence of speed-accuracy trade-offs (i.e., opposite effect signs in RTs and ERs). Error bars represent standard errors.

**FIGURE 4:**
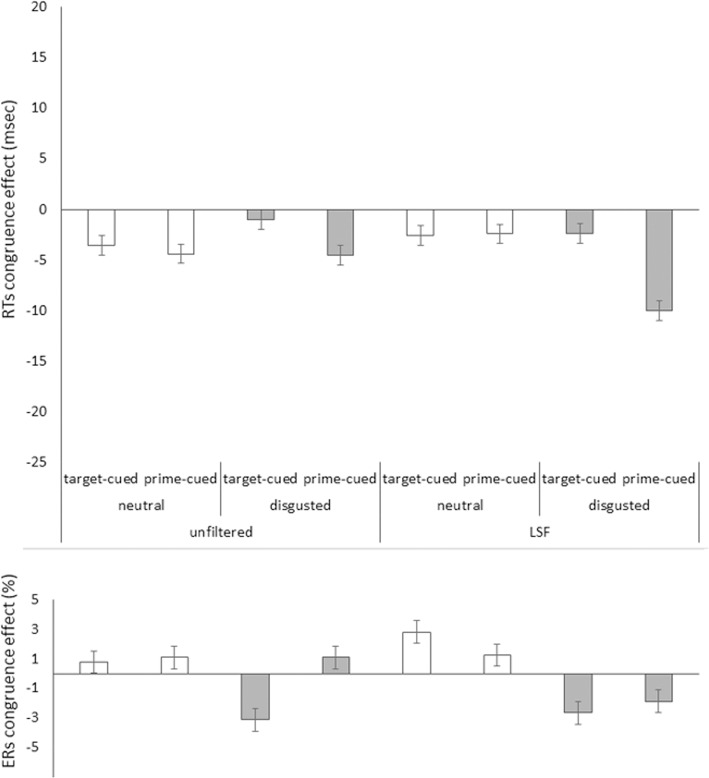
Priming effects (incongruent RTs minus congruent RTs) in mean correct RTs in milliseconds **(Upper)** and in error rates (ERs) in percent **(Lower)** on the *y* axis plotted as a function of prime face filtering (unfiltered vs. LSF filtered primes), target face valence (neutral [white bars] vs. disgusted [gray bars]), and cue type (target-cued vs. prime-cued) in Experiment 1’s LSF block on the *x* axis. Jointly the congruence effects in RTs and ERs show reversed priming effects in the unfiltered target-cued disgusted faces, as well as in both target-cued and prime-cued LSF primes blocks disgusted face target conditions. The remaining conditions apparently show speed-accuracy trade-offs (i.e., opposite effects signs in RTs and ERs). Error bars represent standard errors.

For our first analysis, only correct target responses were considered. Mean correct RTs for each participant and condition were calculated. In order to correct for potential speed-accuracy tradeoffs, we applied the so-called “killing-the-twin” procedure (Grice et al., 1977; Eriksen, 1988) here and throughout the study: Separately for each condition, twins (nearest in magnitude) of erroneous RTs were searched in the correct RTs and discarded (5.6%; Inclusion versus exclusion of twins of the erroneous responses had no qualitative or quantitative impact on the results.) Furthermore, trials that were faster or slower than 2.5 standard deviations of the corrected mean were also discarded (3.8%). Maybe a less biased approach to the removal of outliers could have been based on the interquartile range (IQR), but we preferred to apply the same outlier criterion as was used in prior studies of face priming in this paradigm to rule out that potential differences were due to marginal methodological details. The priming effects in mean correct RTs (incongruent RT minus congruent RT) for all our variables are depicted in **Figure 3**, upper panel, for the HSF block, and in **Figure 4**, upper panel for the LSF block.

An omnibus mixed-measures analysis of variance (ANOVA) was performed, with the within-participant variables target valence (neutral vs. disgusted), cue type (target-cued vs. prime-cued), prime filtering (unfiltered vs. filtered primes), and prime-target congruence (congruent vs. incongruent), as well as the between-participants variable prime frequencies (HSF vs. LSF). For any *post hoc* tests, Bonferroni adjustments for multiple comparisons and the alpha level of 0.05 for all statistics were applied here and throughout the study. See **Table 1** for the results of the ANOVA.

**Table 1:** Results of an analysis of variance (ANOVA) of the mean correct reaction times (RTs), with the within-participant variables target valence (neutral vs. disgusted), cue type (target-cued vs. prime-cued), prime filtering (unfiltered vs. filtered primes), and prime-target congruence (congruent vs. incongruent), as well as the between-participants variable prime frequencies (high spatial frequencies [HSF] vs. low spatial frequencies [LSF]) in the target-discrimination task of Experiment 1.

Effect/Interaction	*F*(1,38)	Sig. *p*	Partial η^2^	Mean RTs (*M* in ms), further *t*-tests and remarks
Cue type	17.48	0.001	0.32	Participants performed faster in the target-cued condition (*M* = 504 ms) than in the prime-cued condition (*M* = 510 ms), laying proof of the expected influence of spatial attention (Finkbeiner and Palermo, 2009; Khalid et al., 2013, 2015).
Congruence	<1.00			The main effect of congruence was non-significant.
Target valence	<1.00			See also the error rates in **Table 2**.
Target-Valence × Congruence interaction	<1.00			We expected a greater congruence effect with the disgusted faces. However, both the main effect of congruence and its interaction with target valence were non-significant. See also the error rates in **Table 2**.
Prime Frequencies × Congruence interaction	5.70	0.02	0.13	Paired *t*-tests showed a significant reverse congruence effect only for the LSF primes, *t*(19) = 2.21, *p* = 0.04. Unexpectedly, participants performed faster in the incongruent (*M* = 511 ms) than in the congruent (*M* = 515 ms) condition. The HSF primes did not show a significant congruence effect (congruent *M* = 500 ms; incongruent *M* = 502 ms), *t*(19) = 1.10, *p* = 0.29. This interaction is important for conclusions about the subcortical origin of the face congruence effect and is indicative of a reversed congruence effect with LSF primes only.
Prime Frequencies × Target Valence × Cue-Type	4.64	0.04	0.11	This was an unexpected interaction. Interested readers should refer to the follow-up ANOVAs (a) and (b) below, where the data was split-up for prime frequencies (HSF and LSF prime blocks) and collapsed across prime filtering and prime-target congruence.
Filtering × Target Valence × Congruence × Spatial Frequencies interaction	<1.00			Non-significant interaction. See also the error rates in **Table 2**.
(a) HSF primes:	*F*(1,19)			
Cue type	7.94	0.01	0.30	Means showed faster responses in the target-cued (*M* = 498 ms) than in the prime-cued (*M* = 503 ms) condition. This effect showed the expected influence of spatial attention.
Target Valence × Cue-Type interaction	5.97	0.02	0.24	Paired *t*-tests showed a significant effect of cue-type only for the neutral targets, *t*(19) = 5.00, *p* < 0.001. Participants performed faster in the target-cued (*M* = 497 ms) than in the prime-cued (*M* = 507 ms) condition. However, the disgusted targets did not show a significant cue-type effect (*M* target-cued = 499 ms; *M* prime-cued = 500 ms), *t*(19) < 1.00. These results showed that performance was facilitated by the cue only with neutral targets and not with disgusted targets.
(b) LSF primes block:	*F*(1,19)			The same ANOVA as for the HSF primes block.
Cue type	9.54	0.01	0.33	Participants performed faster in the target-cued condition (*M* = 510 ms) than in the prime-cued condition (*M* = 517 ms).
Target Valence × Cue-Type interaction	<1.00			Non-significant interaction showing that both the neutral and disgusted targets were facilitated by the cue.

*Bins analysis:* We ran the omnibus ANOVA with the additional variable of bin (1, 2, and 3). Besides the above reported results, this ANOVA additionally showed a tendency toward a significant four-way interaction of bin with prime frequencies, target valence, and congruence, *F*(2,76) = 3.03, *p* = 0.054, partial η^2^ = 0.07. However, follow-up analyses did not show any significant interaction of bin with congruence.

#### Error Rates

See **Figure 3**, lower panel for mean ERs in the HSF block, and **Figure 4**, lower panel for the LSF block. The same omnibus ANOVA as for RTs was also run for the error rates (ERs, total = 5.6%). See **Table 2** for the results of this ANOVA.

**Table 2:** Results of an analogous ANOVA of the error rates (ERs), with the within-participant variables target valence (neutral vs. disgusted), cue type (target-cued vs. prime-cued), prime filtering (unfiltered vs. filtered primes), and prime-target congruence (congruent vs. incongruent), as well as the between-participants variable prime frequencies (high-spatial frequencies [HSF] vs. [LSF]) in the target-discrimination task of Experiment 1.

Effect/Interaction	*F*(1,38)	Sig. *p*	Partial η^2^	Mean ERs (*M* in %), further *t*-tests and remarks
Cue type	7.43	0.01	0.16	*M* target-cued = 6.1%; *M* prime-cued = 5.2%. This effect turned out to be in the opposite direction as compared to the expectations and as compared to the RT effects.
Congruence	1.04	0.31		The main effect of congruence was non-significant.
Target valence	46.91	0.001	0.55	*M* neutral = 4.4%, *M* disgusted = 6.9%. This was an unexpected result.
Target Valence × Congruence interaction	35.61	0.001	0.48	Most important to our hypothesis of a subliminal emotional congruence effect: Mean ERs in the congruent and incongruent conditions differed significantly for the neutral target faces (congruent: *M* = 3.7% vs. incongruent: *M* = 5.2%), *t*(39) = 3.32, *p* < 0.01, and were significantly reversed for the disgusted target faces (congruent: *M* = 8.0% vs. incongruent: *M* = 5.8%), *t*(39) = 4.62, *p* < 0.001.
Prime Frequencies × Congruence interaction	<1.00			See also the RTs in **Table 1**.
Prime Frequencies × Target Valence × Cue-Type	<1.00			See also the RTs in **Table 1**.
Filtering × Target Valence × Congruence × Spatial Frequencies interaction	6.90	0.01	0.15	Important to the hypothesis regarding the subcortical origin of emotion congruence, see follow-up ANOVAs (a) and (b) below.
(a) Unfiltered primes:				To explore the above four-way interaction, follow-up ANOVAs were conducted for the data split-up for the unfiltered and filtered primes.
Target valence	17.22	0.001	0.31	*M* neutral = 4.7%, *M* disgusted = 6.8%. This was an unexpected result.
Target Valence × Congruence interaction	22.12	0.001	0.37	Mean ERs in the congruent and incongruent conditions differed significantly for the neutral target faces (congruent: *M* = 3.8% vs. incongruent: *M* = 5.7%), *t*(39) = 3.23, *p* < 0.01, and were significantly reversed for the disgusted target faces (congruent: *M* = 7.9% vs. incongruent: *M* = 5.8%), *t*(39) = 3.25, *p* < 0.01.
Target Valence × Congruence × Prime Frequencies	5.86	0.02	0.13	To explore this interaction, the following further ANOVAs were conducted for the data split-up for the levels of the variable target face valence, see (a.1) and (a.2) below.
Target Valence × Prime Frequencies	<1.00			
(a.1) Neutral targets/unfiltered primes:				Here only a significant main effect of congruence was found.
Congruence	10.90	0.02	0.22	Participants performed better in the congruent condition (*M* = 3.8%) than in the incongruent condition (*M* = 5.7%).
(a.2) Disgusted targets/unfiltered primes:				The ANOVA for the disgusted targets primed by unfiltered primes also showed a significant but reversed main effect of congruence.
Congruence	11.10	0.01	0.23	This was a reversed congruence effect: participants performed better in the incongruent condition (*M* = 5.8%) than in the congruent condition (*M* = 7.9%).
(b) Filtered primes:				Follow-up ANOVA for the filtered primes, as in (a) above.
Target valence	29.02	0.001	0.43	Mean ERs showed that the participants performed better in the neutral condition (*M* = 4.1%) than in the disgusted condition (*M* = 6.9%).
Target Valence × Congruence interaction	15.79	0.001	0.29	Mean ERs of different congruence conditions differed almost significantly for the neutral target faces (congruent: *M* = 3.6% vs. incongruent: *M* = 4.6%), *t*(39) = 1.89, *p* = 0.07, and were again reversed for the disgusted target faces (congruent: *M* = 8.1% vs. incongruent: *M* = 5.8%), *t*(39) = 3.71, *p* < 0.01.
Target Valence × Congruence × Prime Frequencies	<1.00			
Target Valence × Prime Frequencies interaction	3.60	0.06	0.09	Marginally significant interaction. Mean ERs of different target emotions differed significantly for the HSF primes (neutral: *M* = 3.6% vs. disgusted: *M* = 7.4%, *t*(39) = 5.09, *p* < 0.001) and to a lesser extent for the LSF primes (neutral: *M* = 4.6% vs. disgusted: *M* = 6.5%), *t*(39) = 2.50, *p* < 0.03.

#### Prime Visibility

The participants were not able to successfully discriminate the emotion of the masked primes as either neutral or disgusted with better than chance accuracy. One-sample *t*-tests against a chance-level performance criterion of 50% correct responses were conducted. As these frequentist tests are not optimized for the support of the null hypothesis, we also computed their Bayes Factors (BFs) with their recommended bench mark scale factor R of 1.0 (Rouder et al., 2009). In the current study, we report the scaled JZS (Jeffreys, Zellner, and Siow) BFs. These BFs indicate the ratio of marginal likelihoods for the null versus the alternative hypothesis (or vice versa) (Jeffreys, 1961; Kass and Raftery, 1995). Conventionally, a Bayes factor greater than 3 is considered as substantial evidence in favor of the null or the alternative hypothesis (Jeffreys, 1961; Dienes, 2011). The *t*-tests indicated that the participants performed not significantly different from chance level in the unfiltered primes’ target-cued condition (*M* = 50.1%), *t*(39) = 0.07, *p* = 0.94, *BF* = 8.10 in favor of the null hypothesis, and prime-cued condition (*M* = 48.9%), *t*(39) = 0.98, *p* = 0.33, *BF* = 5.10 in favor of the null hypothesis, as well as in the filtered primes’ target-cued condition (*M* = 51.5%), *t*(39) = 1.21, *p* = 0.23, *BF* = 4.01 in favor of the null hypothesis, and prime-cued condition (*M* = 51.5%), *t*(39) = 1.06, *p* = 0.30, *BF* = 4.72 in favor of the null hypothesis.

#### Unmasked Prime Discrimination

The participants were able to successfully discriminate the emotion of the unmasked primes as either neutral or disgusted with better than chance accuracy. In the HSF block, unfiltered primes (*M* = 95.1%), and filtered primes (*M* = 92.8%) were discriminated with high rates of accuracy, and the same was true of the LSF block: unfiltered primes (*M* = 93.8%), filtered primes (*M* = 92.9%), in all conditions *t*s > 32.00, *p*s < 0.001, with all *BF*s > 1300000.00 in favor of the alternative for tests against chance performance (50%).

### Discussion

The results showed unanimous evidence for reversed priming effects with the disgusted face targets. With the disgusted faces as targets, error rates were lower in incongruent than in congruent conditions with HSF, LSF, and unfiltered primes. A similar effect was found in the RTs of the LSF primes, where the reversed priming effect seemed to extend to the neutral targets. This generalization of the reversed priming effect to the RTs of the LSF primes is the only weak indication of a subcortical origin of the, in this case reversed, priming effect. These effects are surprising in terms of motor activation theory because prior reports would have suggested to expect an advantage in congruent as compared to incongruent conditions (e.g., Neumann and Lozo, 2012). In addition, usually, reversed priming effects in masked priming experiments are more typical of longer prime-target intervals (e.g., Eimer and Schlaghecken, 1998; for a review, see Sumner, 2007). Maybe a strong signaling function of disgusted facial expressions of adverse situations is more compatible with the participants’ quick avoidance or suppression of this situation. If that was the case, target emotion perception in the congruent disgusted condition could have fallen prey to the quick suppression of the disgusted face prime, whereas this suppression could have in turn facilitated responding to the alternative emotionally neutral face category. Another theoretical interpretation in terms of more adaptation to disgusted than to neutral faces does not make much sense. Although adaptation to a facial emotional expression can in principle decrease processing of the same expression in a temporally following face stimulus (Winston et al., 2004), adaptation effects typically occur on a longer time scale (around 5 s) and are not observed with such short adaptor durations and adaptor-target intervals as were realized here, where the prime would have been a potential adaptor. It is also unclear, why an effect of adaptation should have been restricted to the disgusted face targets and would not have extended to the neutral face targets.

In addition to the perplexing reversed priming effect with the disgusted face targets, we found a more straightforward advantage in the error rates of the congruent as compared to the incongruent conditions with the neutral face targets. The only exception to this pattern was the reversed priming effect in the RTs to neutral target faces of the LSF group that implies that the ER priming effect with neutral face targets in the LSF group could have likewise reflected a speed-accuracy trade-off.

Of further interest, we also found evidence of a cueing effect. Responses to the target faces were faster in target-cued than prime-cued conditions. This cueing effect was likely due to attention, facilitating the processing of the target if the cue happened to direct attention to the target position. Also, as the priming effect of the masked primes was not affected by the cueing effect and as participants showed no indication of an awareness of the masked primes, as in prior studies (Finkbeiner and Palermo, 2009; Khalid et al., 2013), the pattern of results is in line with a coincidence of an independence of awareness and of an independence of attention of the processing of the masked primes and, thus, with a classic view of automatic processing of emotional expressions (cf. Posner and Snyder, 1975). One should note, however, that our manipulation of spatial attention was soft and that our cueing procedure could have, thus, spared enough attentional resources for prime processing even under target-cued conditions. Authors have argued for the usage of highly demanding secondary tasks for potentially more conservative tests of attention-independent processing (Lavie, 1995; Pessoa, 2005).

Finally, the control conditions with the unasked primes made clear that each of the primes that we used in the present study carried sufficient information to be correctly discriminated under aware conditions. This finding shows that the differences between the priming effects of neutral and disgusted faces cannot simply be explained by the difficulty with which disgusted versus neutral face primes could be discriminated *per se*.

## Experiment 2

In Experiment 2, we intended to replicate the reversed priming effect of Experiment 1. As filtering had no strong impact on priming effects in Experiment 1, in Experiment 2, we only used unfiltered primes. In addition, we also used a within-participant comparison between the priming effects of disgusted faces and a second class of negative emotional faces, fearful faces. This was done to investigate if the reversed priming effect that we found with masked disgusted faces in Experiment 1 was emotion-specific, or whether it was maybe typical of any negative valence stimulus. To note, both disgusted and fearful faces express emotions of a negative valence, and a valence-based effect should therefore be the same with fearful and with disgusted faces. However, disgusted and fearful faces depend on different underlying processes (Breiter et al., 1996; Anderson et al., 2003; Phillips et al., 2004; for a meta-analysis see Vytal and Hamann, 2010). Thus, it is also well conceivable that the priming effects of disgusted and fearful faces differ from one another. For instance, fearful faces seem to attract attention more than disgusted faces (Khalid et al., 2017), meaning that if the reversed priming effect with the disgusted faces was really due to more avoidance and suppression of these faces, it might not generalize to fearful faces.

### Method

#### Participants

Thirty-five students (26 female) with a mean age of 21.9 years participated. Measurement by the German version of the State-Trait Anxiety Inventory (STAI; Laux et al., 1981) showed that all participants had normal state (*M* = 34.7, *SD* = 6.5) and trait (*M* = 41.6, *SD* = 8.2) anxiety levels. Further, assessment by the German version of the QADP (Schienle et al., 2002) showed that all participants had normal disgust sensitivity (*M* = 2.0, *SD* = 0.4).

#### Apparatus, Stimuli, and Procedure

These were the same as before with the following exceptions. First, all primes were unfiltered and there was, thus, no between-participants variable. Second, for the second block, four fearful faces, two male and two female (mean RTs = 605 ms, mean ERs = 11.7%), were selected from the pilot experiment. As mentioned in Experiment 1, the participants’ performance in the pilot experiment for the disgusted faces was (mean RTs = 604 ms, mean ERs = 11.8%). Thus, we balanced the performance difficulty for the two classes of negative expressions based upon participants’ responses to them (see **Figure 1**). Third, the block order, fearful faces before disgusted faces or the other way round, was balanced across participants. Fourth, all prime-visibility tests were administered at the end of the experiment, again blocked for different emotional expressions, again with tests of masked faces before tests of unmasked faces. Fifth, as in Experiment 1, each block consisted of 32 repetitions of each combination of cueing (prime-cued, target-cued), target emotion (neutral, emotional), and prime-target congruence level (congruent, incongruent), for a total of 256 trials, and, thus, a total of 512 trials for the target-discrimination task. Also the prime discrimination consisted of two blocks, one for the fearful block and one for the disgusted block, with 128 trials per each block, thus, a total of 256 trials. Again there were 80 trials for the unmasked blocks and 32 training trials in the beginning of each of the target-discrimination blocks. Sixth, the task of the participants was to categorize the neutral versus disgusted, or the neutral versus fearful faces. Seventh, the experiment was run in a single session, with eight short breaks, two in the middle and one after each target-discrimination block, plus one after each of the prime-discrimination blocks, taking approximately 1 h in total.

### Results

#### Target-Discrimination Task

Mean correct RTs, devoid of killed twins (4.2%) for erroneous RTs, were filtered by the same RT criterion as in Experiment 1 (leading to the elimination of 6.0% of all trials).

An omnibus repeated-measures ANOVA was run, with the within-participant variables target emotion (disgusted vs. fearful; between blocks), target valence (neutral vs. emotional; within blocks), cue type (target-cued vs. prime-cued; within blocks), and prime-target congruence (congruent vs. incongruent; within blocks). In contrast to Experiment 1, this ANOVA did not show any significant effect of congruence or any significant interaction congruence and any other variable, all *F*s < 3.27, all *p*s > 0.07.

**FIGURE 5:**
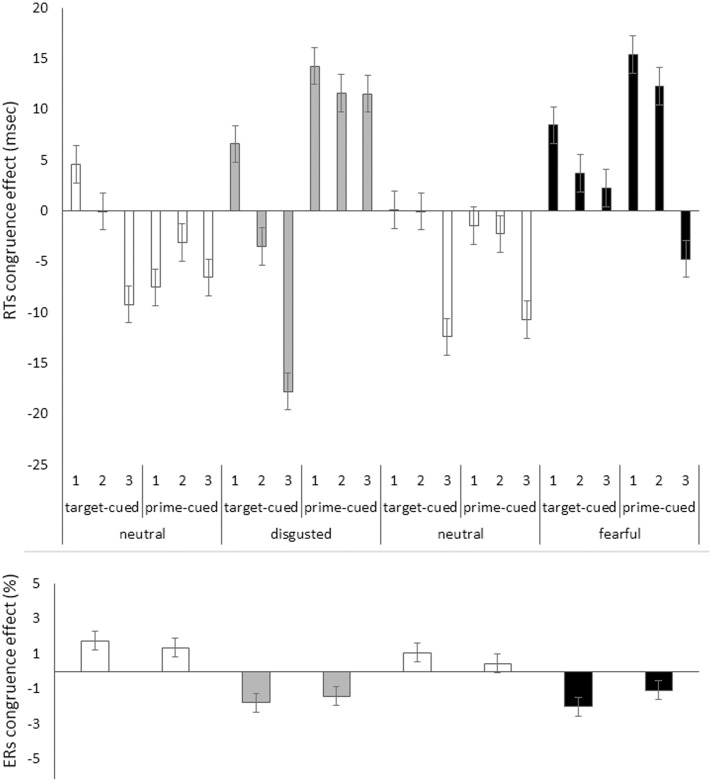
Priming effect (incongruent RTs minus congruent RTs) in mean correct RTs in milliseconds **(Upper)** and in error rates (ERs) in percent **(Lower)** on the *y* axis plotted as a function of bin (1,2, and 3 [only in RTs]), target face emotion type (disgusted vs. fearful block), target face valence (neutral [white bars] vs. disgusted [gray bars] or neutral [white bars] vs. fearful [black bars]), and cue type (target-cued vs. prime-cued) in Experiment 2 on the *x* axis. Jointly the congruence effects in RTs and ERs show reversed priming effects only with the target-cued disgusted face conditions. All other conditions apparently show speed-accuracy trade-offs (i.e., opposite effects signs in RTs and ERs). Error bars represent standard errors.

*Bins analysis:* For an exhaustive analysis, again, RTs were divided into three bins from fast to slow responses. We ran the above omnibus ANOVA with the additional variable of bin (1, 2, and 3; within blocks). In contrast to Experiment 1, RT bin interacted significantly with congruence (see below). The priming effects in mean correct RTs as a function of all our variables are depicted in **Figure 5**, upper panel.

See **Table 3** for the results of the ANOVA.

**Table 3:** Results of an ANOVA of the RTs, with the within-participant variables bin (1, 2, and 3; within blocks), target emotion (disgusted vs. fearful; between blocks), target valence (neutral vs. emotional; within blocks), cue type (target-cued vs. prime-cued; within blocks), and prime-target congruence (congruent vs. incongruent; within blocks) in the target-discrimination task of Experiment 2.

Effect/Interaction	*F*	Sig. *p*	Partial η^2^	Mean RTs (*M* in ms), further *t*-tests, and remarks
Cue type	(1,34) = 7.25	0.01	0.18	RTs were shorter in the target-cued condition (*M* = 523 ms) than in the prime-cued condition (*M* = 528 ms).
Congruence	<1.00			The main effect of congruence was non-significant.
Target valence	(1,34) = 4.03	0.053	0.11	Marginally significant main effect, RTs were shorter in the emotional condition (*M* = 521 ms) than in the neutral condition (*M* = 529 ms).
Target Valence × Congruence interaction	(1,34) = 5.25	0.03	0.13	Mean RTs showed a marginally significant reversed congruence effect for the neutral faces (congruent: *M* = 532 ms vs. incongruent: *M* = 528 ms), *t*(34) = 1.96, *p* = 0.06, but not for the emotional faces (congruent: *M* = 518 ms vs. incongruent: *M* = 523 ms), *t*(34) = 1.67, *p* = 0.11. This was again in accordance with the hypothesis of a (potentially transient) subliminal influence of emotional facial expressions.
Target Valence × Cue Type interaction	(1,34) = 8.41	0.01	0.20	This was unexpected result. Pairwise comparisons showed that the effect of cue type was significant only in the neutral targets condition (target-cued: *M* = 524 ms vs. prime-cued: *M* = 535 ms), *p* < 0.001, but not in the emotional targets condition, *p* > 0.77.
Face emotion	(1,34) = 19.75	0.001	0.35	RTs were shorter in the block with the disgusted faces (*M* = 510 ms) than in the block with the fearful faces (*M* = 540 ms). This result was unexpected.
Bin	(2,68) = 767.00	0.001	0.96	This was a trivial expected RT increase from first to last bin.
Bin × Congruence interaction	(2,68) = 20.78	0.001	0.38	This reflected a significant congruence effect in the 1st bin (congruent: *M* = 446 ms vs. incongruent: *M* = 451 ms), *t*(34) = 2.85, *p* < 0.01, as well as a reversed congruence effect in the 3rd bin (congruent: *M* = 617 ms vs. incongruent: *M* = 611 ms), *t*(34) = 2.60, *p* < 0.01, but no significant congruence effect in the 2nd bin (congruent: *M* = 512 ms vs. incongruent: *M* = 514 ms), *t*(34) = 1.35, *p* = 0.19. This was in accordance with the hypothesis of a (potentially transient) subliminal influence of emotional facial expressions.
Bin × Face Emotion × Cue Type × Congruence interaction	(2,68) = 4.89	0.02	0.13	This interaction was potentially informative to all hypotheses of interest–that is, the attention-independence of the facial congruence effects, their emotion specificity, and their potential temporal transience. This interaction was, therefore, further investigated by follow-up analyses split-up for the variable face emotion (fearful vs. disgusted blocks, see [a] and [b] below).
(a) Fearful target block:				The ANOVA for the discrimination of the fearful versus neutral face targets, with the within-participant variables bin, face valence, cue type, and congruence.
Bin × Congruence interaction	(2,68) = 8.57	0.01	0.20	Means showed significant congruence effects only in the 1st bin (congruent: *M* = 450 ms vs. incongruent: *M* = 462 ms), *t*(34) = 3.54, *p* < 0.01, but neither in the 2nd (congruent: *M* = 521 ms vs. incongruent: *M* = 529 ms), *t*(34) = 1.85, *p* = 0.07, nor in the 3rd bin (congruent: *M* = 631 ms vs. incongruent: *M* = 630 ms), *t*(34) < 1.00. In line with the hypothesis of a temporally transient congruence effect of the subliminal face primes, these *post hoc* tests showed a congruence effect only in the fastest responses but not in the slower responses.
Bin × Congruence × Cue Type interaction	<1.00			
(b) Disgusted target block:				The ANOVA for the discrimination of the disgusted versus neutral face targets with variables as in (a) above.
Bin × Congruence interaction	(2,68) = 6.49	0.01	0.16	See the following follow-up ANOVAs, (b.1) and (b.2).
Bin × Congruence × Cue Type interaction	(2,68) = 4.12	0.03	0.11	To further explore these interactions, follow-up ANOVAs were conducted for the data from the disgusted target block split-up for the variable cue type (prime-cued vs. target-cued, see [b.1] and [b.2] below).
(b.1) Disgusted target block, prime-cued:				With the within-participant variables bin, face valence, and congruence.
Bin × Congruence interaction	<1.00			
Target Valence × Congruence interaction	(2,68) = 4.89	0.03	0.13	Means showed a significant congruence effect only with the disgusted target faces (congruent: *M* = 499 ms vs. incongruent: *M* = 511 ms), *t*(34) = 2.11, *p* < 0.04, but not with the neutral target faces (congruent: *M* = 525 ms vs. incongruent: *M* = 519 ms), *t*(34) = 1.17, *p* = 0.25.
(b.2) Disgusted target block, target-cued:				Again with the within-participant variables bin, face valence, and congruence.
Bin × Congruence interaction	(2,68) = 9.84	0.01	0.23	Means showed neither a significant congruence effect in the 1st bin (congruent: *M* = 431 ms vs. incongruent: *M* = 437 ms), *t*(34) = 1.60, *p* = 0.12, nor in the 2nd bin (congruent: *M* = 495 ms vs. incongruent: *M* = 494 ms), *t*(34) < 1.00, but a significantly reversed congruence effect in the 3rd bin (congruent: *M* = 597 ms vs. incongruent: *M* = 583 ms), *t*(34) = 2.95, *p* < 0.01.
Target Valence × Congruence interaction	<1.00			

#### Error Rates

The same omnibus ANOVA was conducted on ERs (4.2% in total). See **Table 4** for the results of the ANOVA.

**Table 4:** Results of ANOVA of the mean error rates (ERs), with the within-participant variables target emotion (disgusted vs. fearful; between blocks), target valence (neutral vs. emotional; within blocks), cue type (target-cued vs. prime-cued; within blocks), and prime-target congruence (congruent vs. incongruent; within blocks) in the target-discrimination task of Experiment 2.

Effect/Interaction	*F*(1,34)	Sig. *p*	Partial η^2^	Mean ERs (*M* in %), further *t*-tests, and remarks
Cue type	7.56	0.01	0.18	Unexpectedly participants made more errors in the target-cued condition (*M* = 4.5%) than in the prime-cued condition (*M* = 3.8%).
Congruence	<1.30			The main effect of congruence was non-significant.
Target valence	41.17	0.01	0.55	Unexpectedly participants made more errors with the emotional faces (*M* = 5.1%) than with the neutral faces (*M* = 3.2%).
Target Valence × Congruence interaction	23.35	0.01	0.41	This was of major interest for our hypothesis of a subliminal emotional congruence effect and was explored further in follow-up ANOVAs, with data split for target emotions, see [a] and [b] below).
Target Valence × Cue Type interaction	10.74	0.01	0.24	As in RTs this was an unexpected result, but see follow-up ANOVAs, (a) and (b) below.
Face emotion	<1.00			The main effect of face emotion was non-significant.
(a) Emotional faces:				with the within-participant variables target face emotion (disgusted vs. fearful), cue type (target-cued vs. prime-cued), and congruence (congruent vs. incongruent).
Congruence	24.73	0.01	0.42	After Experiment 1, we expected a reversed congruence effect for the disgusted faces. This was true: Participants made more errors in the congruent condition (*M* = 5.9%) than in the incongruent condition (*M* = 4.3%).
Cue type	13.38	0.01	0.28	As in the RTs, unexpectedly, participants made more errors in the target-cued condition (*M* = 5.8%) than in the prime-cued condition (*M* = 4.4%).
(b) Neutral faces:				Analogous to the emotional faces’ ANOVA.
congruence	10.29	0.01	0.23	This was due to a conventional congruence effect: Participants made more errors in the incongruent condition (*M* = 3.8%) than in the congruent condition (*M* = 2.6%).
Cue type	<1.00			The neutral target faces were not facilitated by the cue.

#### Prime Visibility

The participants were once again not able to successfully discriminate the emotion of the masked primes as either neutral or disgusted with better than chance accuracy. One-sample *t*-tests against chance level accuracy (50%) indicated that the participants performed not significantly different from chance level performance in the disgusted block, target-cued condition (*M* = 51.4%), *t*(34) = 0.79, *p* = 0.44, *BF* = 5.63 in favor of the null hypothesis, and prime-cued condition (*M* = 52.9%), *t*(34) = 1.69, *p* = 0.10, *BF* = 1.99 in favor of the null hypothesis (this latter value is less than 3, and therefore not substantial evidence), as well as in the fearful block, target-cued condition (*M* = 48.2%), *t*(34) = 1.19, *p* = 0.24, *BF* = 3.87 in favor of the null hypothesis, and prime-cued condition (*M* = 52.2%), *t*(34) = 1.77, *p* = 0.09, *BF* = 1.76 in favor of the null hypothesis (again, this latter value is less than 3, and therefore not substantial evidence).

#### Unmasked Prime Discrimination

The participants were able to successfully discriminate the emotional expressions of the unmasked prime faces in both unmasked disgusted faces (*M* = 92.3%), *t*(34) = 37.76, *p* < 0.001, *BF* > 1200000.00 in favor of the alternative hypothesis, and unmasked fearful faces (*M* = 89.1%,), *t*(34) = 26.24, *p* < 0.001, *BF* > 1100000.00 in favor of the alternative hypothesis. Moreover, the discrimination of the unmasked disgusted primes was significantly better than that of the fearful primes, *t*(34) = 2.82, *p* < 0.01, *BF* = 4.30 in favor of the alternative hypothesis.

### Discussion

The results of Experiment 2 showed a reversed priming effect in the ERs for both emotional facial expressions that we used. This was in contrast to the ER effects with neutral faces that showed a more typical priming effect, with advantages in congruent as compared to incongruent conditions. Moreover, only with the target-cued disgusted faces, the reversed priming effect was also found in the RTs. In all other conditions, RTs showed some evidence of an RT priming effect that was in the opposite direction of the respective ER priming effect: With prime-cued disgusted target faces and with fearful target faces, RTs were faster in congruent than incongruent conditions, while with neutral target faces, RTs were faster in incongruent than congruent conditions. In conclusion, we only found clear-cut evidence of a reversed priming effect with target-cued disgusted face targets, whereas all other priming effects showed signs of speed-accuracy trade-offs. Please also note that the priming effects were non-significant in the initial RTs analysis, but were revealed only in the subsequent bins analysis and in the ERs.

What might have caused the dependence of the reversed priming effect on attention is unclear. This finding, at variance with what was observed in Experiment 1, shows that the classical criteria of automatic processing, such as awareness-independence and attention-independence, do not necessarily always converge (Kahneman and Treisman, 1984; Bargh, 1994; Moors and De Houwer, 2006). Maybe the reverse priming effect indeed reflected some type of suppression of the prime content that was here further exacerbated by the target-directed attention elicited by the cues. The fact that this was not found in Experiment 1, however, suggests that simple group differences between the participants of the two experiments might have played a role, too. In any case, one should bear in mind that the current manipulation of spatial attention might not have been the strongest measure of depleting attentional resources at the expense of prime processing, so that future studies should try to investigate the role of attention for (reverse) subliminal priming effects also with alternative means (e.g., highly demanding secondary tasks; cf. Lavie, 1995; Pessoa, 2005). In fact, in the current experiment, there was not much evidence for the intended attentional manipulation in the first place, as the cueing effects were in an unexpected direction.

With respect to the priming effects of all other conditions, primes used with fearful face targets, primes used with neutral face targets, and prime-cued primes used with disgusted face targets, the results were disappointing as no clear priming effects could be found. This finding is disappointing because at least motor priming should have fostered the priming effect: The primes were used as targets and carried a meaning that fitted to the participants’ task of a categorical decision between neutral and emotional faces. These would have been ideal conditions for an awareness-independent motor priming effect (Klotz and Neumann, 1999; Kunde et al., 2003). The fact that the masked and, thus, liminal primes were without effect is, however, in line with the claim that little processing of emotional facial expressions occurs outside awareness, once physical stimulus differences are controlled for Hedger et al. (2015). This is exactly what we have done in the current study: We have used faces of different emotional expressions as primes that were devoid of spectral power and luminance differences between different emotional expressions, thus, probably curtailing differential effects that any of the masked faces might have had on target recognition. This finding is at least in line with the efficiency of our masking procedure, reflected in chance performance in the mask discrimination task, with the exception of the prime-cued conditions in both disgusted and fearful faces blocks, where the Bayes factors did not show substantial evidence in favor of the null hypothesis.

In addition to these effects, we also again found that the unmasked primes contained sufficient information to also successfully discriminate between fearful and neutral face primes.

## General Discussion

In light of past research, the current study asked two questions: Is there any evidence for subcortical visual processing of subliminal facial expressions of disgust, and can we find evidence for the emotion-specificity of the processing of subliminal disgusted faces? Question 1 was addressed in Experiment 1. Subliminal emotional face processing seems to reflect humans’ sensitivity to highly useful visual input that could have been of help in repeatedly overcoming the same threats to human survival in the course of evolution (Öhman and Mineka, 2001; Öhman, 2002). Such sensitivity has the chance to prevent harm and, thus, increase inclusive fitness among those showing this phenotypic trace, for instance, by an increased sensitivity to sources of danger and the possibility to avoid them among those who inherited a proneness to the detection of fearful human facial displays by genetic predisposition (Öhman, 2002). As researchers have repeatedly linked this hypothesis to the evidence for the subcortical visual processing of a variety of subliminal emotional expressions that are different from disgust (Dolan et al., 1996; Whalen et al., 1998; Jolij and Lamme, 2005; Tamietto and de Gelder, 2010), we tested the possibility that subliminal processing of disgusted faces, equally pointing to potential sources of threat in the environment, could also be carried out along the subcortical visual pathway. For this test, we took advantage of the known exclusive sensitivity of the subcortical visual pathway to LSF content (Schiller et al., 1979; Merigan and Maunsell, 1993; Deruelle and Fagot, 2005; de Gardelle and Kouider, 2010; Khalid et al., 2013). We used differently filtered images of disgusting faces as primes and reduced their visibility to subliminal processing levels by backward masking. We tested if priming effects of subliminal faces are restricted to LSF primes and unfiltered primes, but cannot be found with subliminal HSF primes that are known to not be processed along the subcortical pathway. In Experiment 1, we did not find any evidence for subcortical processing of disgusted faces, as it did not matter if an LSF or HSF prime was used for the face primes.

Also, to our surprise, the only evidence of subliminal processing of disgusted faces took the form of a reversed priming effect, with slower RTs and more errors in conditions in which a subliminal disgusted face prime preceded a supraliminal disgusted face target as compared to a condition with a subliminal emotionally neutral face prime preceding the same disgusted face target. This was surprising because, based on the existing literature, we would have expected the opposite: better performance with congruently than incongruently primed emotional targets (e.g., Neumann and Lozo, 2012). Maybe the reversed priming effect reflected efficient avoidance and suppression of the disgusted faces–a response that seems to be reasonable in terms of the most fruitful and reinforcing behavioral consequences that should be triggered by a disgust-signaling stimulus (Oaten et al., 2009).

The second question was studied in Experiment 2. In Experiment 2, we intended to replicate the surprising inverse priming effect with disgusted face targets and at the same time wanted to test if this effect is emotion-specific. To that end, we compared subliminal priming effects with disgusted face targets to that with fearful face targets within the same participants. In this experiment, little evidence of subliminal emotional processing was found, but the few indications that the subliminal prime faces were processed were again showing a reversed priming effect with the disgusted face targets. Different from Experiment 1, however, in Experiment 2 the reverse priming effect was restricted to conditions in which a cue would have distracted attention away from the primes. Maybe this finding reflected that indeed the avoidance and suppression of the processing of the disgusted faces was responsible for the reverse priming effect and that this suppression was supported by a cue away from the prime.

To note, in both experiments, we had used pre-cues to direct the participants’ attention to either the targets or the primes. This was done in an attempt to test if subliminal processing of emotional displays of faces was also independent of attention (cf. Finkbeiner and Palermo, 2009). Yet, the evidence for an attention-independence of the subliminal emotional face processing was restricted to Experiment 1, as implied by the interaction of cueing and priming with the disgusted faces in Experiment 2, and we do not know why the cues should have exerted their effects on subliminal processing of the disgusted faces in Experiment 2 only. Be that as it may, jointly the results seemed to support the notion that different criteria of automatic processing do not necessarily converge (Bargh, 1994). However, on a more cautionary note, the current manipulation of spatial attention might have been simply too weak to really deplete attentional resources. In line with this assumption, in Experiment 2, the manipulation of attention did not work as intended. Thus, future studies should use more drastic measures, such as a more demanding secondary task, to investigate if subliminal facial priming effects are indeed independent of attention (cf. Pessoa, 2005).

What was also striking was the absence of any clear-cut evidence for subliminal priming effects of the fearful face primes in Experiment 2. This was in contrast with previous studies (e.g., Dolan et al., 1996) and might have reflected the particular care that we took in creating physically equal face prime energies for the different emotional displays. Prior research had already suggested that evidence for the (differential) processing of subliminal emotions could owe to the confounded physical energy differences between different emotional displays (Hedger et al., 2015), so that it may not be such a wonder that our emotional faces, which were equated for their frequency spectra, were not creating much of a subliminal priming effect. In fact, this consideration should also be taken to put the reverse priming effect of the subliminal disgusted faces into perspective: Maybe the reverse priming effect reflected that bit of a net priming effect with subliminal emotional expressions that survived the elimination of the physical differences between different face expressions.

What can hardly be doubted following our experiments is that the face primes contained sufficient information for successful discrimination of different emotional expressions. This conclusion was supported by the participants’ performance in trials in which the same faces that were used as subliminal primes were presented supraliminally. Under supraliminal conditions, participants had little difficulty to tell different emotional face primes from one another.

## Conclusion

Subliminal priming by disgusted faces was found but took the unusual form of reversed priming. This effect seemed to be emotion-specific rather than reflecting the valence of the disgusted faces, as no such processing was found with subliminal faces of a different emotion (i.e., fearful faces). Yet, no evidence for subcortical visual processing of subliminal disgusted faces could be found. From what we know, it could well be that subliminal processing of disgusted faces occurs along the retino-geniculate pathway, maybe even at cortical face processing sites (Kouider et al., 2009; Smith, 2012).

## Ethics Statement

This study was carried out in accordance with the recommendations of APA standards and the rules of the declaration of Helsinki, ethics committee of the University of Osnabrück with written informed consent from all subjects. All subjects gave written informed consent in accordance with the Declaration of Helsinki. The protocol was approved by the ethics committee of the University of Osnabrück.

## Author Contributions

Both authors, SK and UA, substantially contributed to the conception and design of the work, acquisition, analysis, and interpretation of data for the research, as well as drafting the manuscript. Both authors approved the final version for publication, and both agree to be accountable for all aspects of the work in ensuring that questions related to the accuracy or integrity of any part of the work are appropriately investigated and resolved.

## Conflict of Interest Statement

The authors declare that the research was conducted in the absence of any commercial or financial relationships that could be construed as a potential conflict of interest. The reviewer MR and handling Editor declared their shared affiliation, and the handling Editor states that the process nevertheless met the standards of a fair and objective review.
